# *In Vitro* antileishmania activity of sesquiterpene-rich essential oils from *Nectandra* species

**DOI:** 10.1080/13880209.2017.1407803

**Published:** 2017-11-29

**Authors:** Lauriane Serpa Silva Bosquiroli, Ana Caroline dos Santos Ferreira, Katyuce Souza Farias, Eduarda Carneiro da Costa, Maria de Fátima Cepa Matos, Mônica Cristina Toffoli Kadri, Yasmin Silva Rizk, Flávio Macedo Alves, Renata Trentin Perdomo, Carlos Alexandre Carollo, Carla Cardozo Pinto de Arruda

**Affiliations:** a Laboratório de Parasitologia Humana, Centro de Ciências Biológicas e da Saúde, Universidade Federal de Mato Grosso do Sul, Campo Grande, Brasil;; b Laboratório de Produtos Naturais e Espectrometria de Massas – LaPNEM, Centro de Ciências Biológicas e da Saúde, Universidade Federal de Mato Grosso do Sul, Campo Grande, Brasil;; c Laboratório de Biologia Molecular e Culturas Celulares, Centro de Ciências Biológicas e da Saúde, Universidade Federal de Mato Grosso do Sul, Campo Grande, Brasil;; d Laboratório de Biofisiofarmacologia, Centro de Ciências Biológicas e da Saúde, Universidade Federal de Mato Grosso do Sul, Campo Grande, Brasil;; e Herbário CG-MS, CCBS, Universidade Federal de Mato Grosso do Sul, Campo Grande, Brasil

**Keywords:** *Leishmania infantum*, *Leishmania amazonensis*, natural products, cytotoxicity, nitric oxide

## Abstract

**Context:** New antileishmanias are needed because of toxicity, high cost and resistance problems associated with available drugs. *Nectandra* (Lauraceae) produces several classes of compounds but its essential oil has not previously been reported to have antileishmania activity.

**Objective:** We evaluated the cytotoxicity and antileishmania activity of essential oils from *Nectandra amazonum* Nees, *N. gardneri* Meisn., *N. hihua* (Ruiz & Pav.) Rohwer and *N. megapotamica* (Spreng.) Mez.

**Materials and methods:***Nectandra* oils were extracted from stem bark/leaves by hydrodistillation and compounds were identified by GC-MS. Oils were tested against *Leishmania infantum* and *L. amazonensis* intracellular amastigotes and nitric oxide production was evaluated. Cytotoxicity was achieved on NIH/3T3 and J774.A1 cells for the selectivity index (SI).

**Results and discussion:***Nectandra gardneri* was active against *L. infantum* and *L. amazonensis* (IC_50_ =  2.7 ± 1.3/2.1 ± 1.06 μg/mL) and contained 85.4% sesquiterpenes, of which 58.2% was intermediol. Besides low cytotoxicity (SI >11.3), *N. gardneri* induced a significant increase in NO production by *L. infantum*-infected macrophages. *Nectandra hihua* had the best activity on *L. infantum* amastigotes (IC_50_ =  0.2 ± 1.1 μg/mL). This oil was 89.0% sesquiterpenes, with 28.1% bicyclogermacrene. The two specimens of *N. megapotamica* had different activities on amastigotes. The one richer in sesquiterpenes (49.9%) was active against both species (IC_50_ =  12.5 ± 1.4/21.3 ± 1.2) and had phenylpropanoid *E*-asarone as the main compound (42.4%). *Nectandra amazonum* showed moderate activity on both the species (IC_50_ =  31.9 ± 2.0/22.1 ± 1.3 μg/mL) and low selectivity (0.9 < SI >2.6), probably due to the major presence of β-caryophyllene (28.5%).

**Conclusions:** Our data identify compounds that can now be isolated and used for the development of new antileishmanias.

## Introduction

Leishmaniases are a group of vector-borne diseases caused by *Leishmania* protozoan parasites; 350,000,000 people live in areas at risk of acquiring these diseases. Clinical manifestations include the visceral and cutaneous forms of the disease, which together cause nearly 1.6 million cases per year (Alvar et al. 2012). Despite this, leishmaniases have been neglected and excluded from the tropical disease priorities, and relegated to limited therapeutic options.

Novel antileishmanias are needed because the currently available drugs have limited usefulness due to toxicity, high cost and parasite resistance (Freitas-Junior et al. 2012). Natural products are a potential source of new therapeutic agents against leishmaniases (Brahmachari 2012).

The family Lauraceae includes 52 genera distributed mainly in Southeast Asia and Americas (Romoff et al. 2010). *Nectandra* Rol. Ex Rottb is the second genus according to the number of species in the New World (Rohwer 1993). In the Americas, these woody plants are mainly distributed in tropical and subtropical areas (Rohwer and Kubitzki 1993). Some species are used in folk medicine as antifungal, antidiarrhoeal, analgesic and antirheumatic agents (Melo et al. 2006). Studies have shown that extracts of *Nectandra* Rol. ex Rottb species have biological activities, such as antitumour (Le Quesne et al. 1980) and antimalaria (Bohlke et al. 1996; Muñoz et al. 2000) activity.

Several classes of compounds have been described in *Nectandra* spp., such as alkaloids (Santos Filho and Gilbert 1975), phenylpropanoids (Garcez et al. 2009), sesquiterpenes (Romoff et al. 2010) and lignoids (Gottlieb 1972). Antileishmania activity (Silva-Filho et al. 2008) has been described for some lignans and neolignans (Le Quesne et al. 1980; MacRae and Towers 1984; Silva-Filho et al. 2004). Despite this, there have been no studies addressing the antileishmania activity of essential oils from *Nectandra* species.

Studies have described the inhibitory activity of essential oils on human parasites (reviewed by Sharifi-Rad et al. 2017), and recently on *Leishmania*, providing a promising source of new drugs. Here, we aimed to characterize the essential oils from *N. amazonum* Nees, *N. gardneri* Meisn., *N. hihua* (Ruiz & Pav.) Rohwer and *N. megapotamica* (Spreng.) Mez, and to evaluate their cytotoxicity and activity against *Leishmania* (*Leishmania*) *infantum* and *L.* (*L.*) *amazonensis.* These parasites are the respective aetiological agents of visceral and cutaneous leishmaniasis (VL and CL) in the Americas (Ready 2014).

## Materials and methods

### Plant material and extraction of essential oils

Three species of *Nectandra* were collected from different regions of Mato Grosso do Sul (MS) State, Brazil. *Nectandra gardneri* was collected in a Cerrado area in the county of Campo Grande (20°30′8.23″S 54°36′45,5″W). *Nectandra megapotamica* (samples 1 and 2) were collected in different regions of the same county (20°30′16,191″S 54°23′25,026″W and 20°27′11,84″S 54°35′41,20″W, respectively). *Nectandra hihua* was collected in the county of Maracaju (21°43′55.34″S 55°30′29.98″W). *Nectandra amazonum* was collected in a ciliary forest in the county of Cáceres, Mato Grosso State, Brazil (16°82′30.64″S 57°55′80.28″W). All plants were collected between October and November 2013. After identification by Dr Flavio Macedo Alves and Dr Geraldo Alves Damasceno Junior (Botany Laboratory, CCBS/UFMS), voucher material was deposited in the CGMS/UFMS herbarium (Table 1).

**Table 1.** Collection and extraction of essential oils from *Nectandra* spp.

Species (Abbreviation)	Identification of collect	Plant part	Yield %
*N. amazonum* (NAEO)	G.A. Damasceno-Junior 5296	Leaves	0.044%
*N. gardneri* (NGEO)	Alves, F.M. 597	Stem bark	0.032%
*N. hihua* (NHEO)	Alves, F.M. 599	Leaves	0.121%
*N. megapotamica* - 1 (NMEO1)	Alves, F.M. 601	Stem bark	0.153%
*N. megapotamica* - 2 (NMEO2)	Alves, F.M. 598	Stem bark	0.670%

Fresh material (stem bark or leaves) from *Nectandra* specimens (Table 1) was milled and essential oils were extracted by hydrodistillation in a Clevenger apparatus (Vidrolex) for 5 h. Oils were dried with anhydrous sodium sulphate (Vetec, Rio de Janeiro, Brazil) and yields are shown in Table 1.

### Analysis of the essential oils

The oils were prepared in dichloromethane at the concentration of 1 mg/mL, then subjected to a gas chromatography-mass spectrometry (GC-MS), Shimadzu model QP2010 plus with an auto-injector AOC-20i and an RTX-5MS capillary column (30 m × 0.25 mm ×0:25 μm). Nitrogen was applied as carrier gas (flow rate of 1.13 mL/min), with the following temperature program: 75 °C for 6 min, 170 °C for 5 min; and 260 °C for 15 min. The injector temperature was set at 250 °C, and the mass spectra were obtained by electron impact at 70 eV. Constituents were confirmed by comparison with libraries (NIST and WILEY) and calculation of the Kovats index, accepting variations under 30.

### Parasites

Standard strains of *L. infantum* (MHOM/BR/1972/BH46) and *L. amazonensis* (IFLA/BR/1967/PH8) were used for *in vitro* antileishmania assays. Amastigote forms were routinely isolated from infected Golden hamsters (*Mesocricetus auratus*) and maintained as promastigotes in Schneider’s Insect Medium (Sigma-Aldrich^®^) supplemented with 20% foetal bovine serum (Sigma-Aldrich^®^) and 140 μg/mL gentamicin (Sigma-Aldrich^®^) at 26 °C. On the seventh day of cultivation, promastigote forms from up to four serial passages after isolation were used in the experiments.

### Animals

Peritoneal cells used in *in vitro* tests were obtained from BALB/c mice aged eight weeks. The animals were obtained from the central animal facility of the Center for Biological and Health Sciences (CCBS) of the Federal University of Mato Grosso do Sul (UFMS, Brazil) in good health and free of infections or parasites common to rodents, maintained in individually ventilated cages equipped with mini-isolators, fed a balanced feed (Nuvilab CR-1, Nuvital) with free access to water. The study received approval from the local Animal Experimentation Ethical Committee – CEUA/UFMS (protocol 432/2012).

### Activity against intracellular amastigotes

Peritoneal macrophages were isolated from BALB/c mice and placed in a 24-well plate (1 × 10^5^ cells/well in RPMI 1640 medium supplemented with 10% foetal bovine serum and 140 μg/mL gentamicin, Sigma-Aldrich) as described by Bosquiroli et al. (2015). Cells were infected with *L. infantum* or *L. amazonensis* promastigotes (1 × 10^6^ cells/well) and incubated at 35 °C/5% CO_2_ overnight. The oils were added at concentrations of 6.25 to 50 μg/mL in sets of sextuplicate experiments, and cells were incubated at 35 °C/5% CO_2_ for 24 h. Untreated infected cells and amphotericin B (Amphotericin B solution, 250 μg/mL, Sigma) were used as a negative and positive control, respectively. Percentage of infected macrophages and a total number of amastigotes were determined by counting 200 cells sixfold. The infection index was determined by multiplying the percentage of macrophages that had at least one intracellular parasite by the mean number of amastigotes per macrophage, according to Paladi et al. (2012). The half maximum inhibitory concentration (IC_50_) was calculated by nonlinear dose-response regression curve (GraphPad Prism 5.0^®^ software). Results were expressed as the mean ± standard deviation (SD), and the data were analyzed using the Student’s *t*-test. Differences were considered significant at *p* < 0.05 (represented by an asterisk).

### Nitric oxide (NO) evaluation

NO production by infected peritoneal cells was evaluated in the aforementioned cultures according to Costa et al. (2016). Briefly, supernatants (100 µL) were collected 24 h after treatment and incubated with an equal volume of Griess Reagent (1% sulfanilamide/0.1% (Naphthyl) ethylenediamine in 5% phosphoric acid) at room temperature for 10 min. The accumulation of nitrite (NO_2_^−^) was quantified, and the absorbance was determined at 540 nm (Ding et al. 1988). Absorbance was converted to micromoles of NO_2_^−^ by comparing the samples with a standard curve obtained with known concentrations of sodium nitrite (1–10 µM) diluted in RPMI medium (Sigma). Results were expressed as the mean ± standard deviation (SD). Data were analyzed using the Student’s *t*-test, and differences were considered significant at *p* < 0.05 (represented by an asterisk).

### Cytotoxicity assay

Murine macrophage (J774.A1) and fibroblast (NIH/3T3) cells purchased from the Rio de Janeiro Cell Bank (Brazil) were treated with the essential oils (0.25–250 μg/mL) in triplicate to estimate IC_50._ Oils were dissolved in DMSO (Dimethyl Sulfoxide) and diluted in RPMI 1640 medium supplemented with 10% foetal bovine serum (Sigma), 100 U/mL penicillin, 0.1 mg/mL streptomycin and 0.25 μg/mL amphotericin B. The highest concentration of DMSO was 0.25%, and did not affect cell viability. Amphotericin B (0.025 to 25 μg/mL) and cells in culture medium were used as a positive and negative control, respectively. Cell viability was determined using the sulforhodamine B assay (Skehan et al. 1990). Percentage of growth of each test-sample was calculated as described by Monks et al. (1991). IC_50_ was determined by nonlinear regression (Microcal Origin 6.0). Selectivity index (SI) was calculated according to Monzote et al. (2010).

## Results and discussion

Here we describe the antileishmania activity of essential oils obtained from four species of *Nectandra,* a genus with a significant infrageneric differentiation (Rohwer 1993). *Nectandra amazonum*, *N. gardneri*, *N. hihua* and *N. megapotamica* belong to four different informal groups (Damasceno Júnior et al. 2000; Pott et al. 2006; Alves and Ishii 2007) and stand out among the eight species of *Nectandra* occurring in semi-deciduous and tropical alluvial Brazilian rainforests (Alves and Sartori 2009).

The oil from *N. hihua* (NHEO) was the most active on *L. infantum* intracellular amastigotes (IC_50_ value of 0.2 ± 1.1 µg/mL), being more potent than the reference drug amphotericin B (Table 2). The selectivity index (SI) values were 249.4 and 149.0 for fibroblasts and murine macrophages, respectively, reflecting its highly selective action on amastigote forms. NHEO was composed mainly by sesquiterpenes (89%), with a major presence of bicyclogermacrene (28.1%) (Table 3). This compound has been previously associated with the antileishmania activity described for the volatile oils from *Annona coriacea* Mart. (Siqueira et al. 2011) and *A. foetida* Mart. (Costa et al. 2009). The second main compound in NHEO was germacrene D (13.8%). Machado et al. (2012) found this sesquiterpene to be the most representative compound of *Lantana camara* L. essential oil, which was moderately active on *L. chagasi* (= *L. infantum*) promastigotes. Interestingly, NHEO was not quite active on *L. amazonensis* (IC_50_ value of 24.2 ± 1.2 µg/mL), what suggests different susceptibilities of these two species.

**Table 2.** Effect of essential oils from *Nectandra* spp. on *Leishmania* (*Leishmania*) *infantum* and *L. (L.) amazonensis* intracellular amastigotes, cytotoxicity on mammalian cells, and selectivity index.

	*L. infantum* amastigotes	*L. amazonensis* amastigotes	NIH/3T3 cells		J774.A1 cells	
Test-sample	IC_50_ (μg/mL)^a^	IC_50_ (μg/mL)	IC_50_ (μg/mL)^a^	SI^b^	IC_50_ (μg/mL)^a^	SI^b^
*N. amazonum*	31.9 ± 2.0	22.1 ± 1.3	58.0 ± 2.6	1.8/2.6	29.4 ± 1.5	0.9/1.3
*N. gardneri*	2.7 ± 1.3	2.1 ± 1.06	51.6 ± 5.4	19.5/25.0	29.9 ± 0.9	11.3/14.6
*N. hihua*	0.2 ± 1.1	24.2 ± 1.2	54.9 ± 6.1	249.4/2.3	29.8 ± 2.0	149.0/1.2
*N. megapotamica* (1)	>50.0 ± 1.3	19.0 ± 1.3	162.3 ± 12.7	–/8.6	221.6 ± 18.0	–/11.7
*N. megapotamica* (2)	12.5 ± 1.4	21.3 ± 1.2	252.6 ± 8.0	20.2/11.9	415.6 ± 50.0	33.2/19.5
Amphotericin B	0.3 ± 1.1	0.2 ± 1.1	2.2 ± 0.1	6.6/12.2	4.3 ± 0.9	14.3/21.5

a IC_50_: concentration that inhibits 50% of intracellular amastigotes/cellular growth.

b SI: selectivity index: IC_50_ on mammalian cells/IC_50_ on intracellular amastigotes.

The data are representative of three independent experiments.

**Table 3.** Chemical composition (%) of essential oils from *Nectandra* spp.

N.	Compound class	*N. amazonum*	*N. gardneri*	*N. hihua*	*N. megapotamica* 1	*N. megapotamica* 2
	*Monoterpenes (%)*	0.0	0.0	0.0	13.9	0.0
1	Azulene				0.1	
2	Camphene				0.3	
3	Terpinene-4-ol				0.1	
4	α-Limonene				0.9	
5	α-Pinene				8.5	
6	β-Myrcene				0.4	
7	β-Pinene				3.6	
	*Sesquiterpenes (%)*	90.1	85.4	89.0	19.1	49.9
8	Agarospirol		4.0			0.7
9	Alloaromadendrene			1.5		
10	Aromadendrene	3.6				
11	Bergamotene					0.7
12	Bicycloelemene			1.4		
13	Bicyclogermacrene			28.1		
14	β-Caryophyllene	28.5		9.0		
15	Cedren-3-ol					1.9
16	Cubenol		0.63		2.2	1.4
17	Cyclosativene				0.2	
18	Eremophilene		2.1	1.4		
19	Germacrene B	14.8		3.0		
20	Germacrene D	2.2	3.5	13.8	0.7	0.8
21	Germacrene-D-*4*-ol					0.5
22	Guaiene	2.4				
23	Guaiol		0.9			
24	Humulene epoxide II				0.3	
25	Intermediol	16.2	58.2			
26	Rosifoliol	0.6		1.2		
27	Sabinene				0.4	
28	Selinene	3.8				
29	Spathulenol	0.9	0.6	6.0	0.5	
30	Viridiflorol	0.5	0.8	4.2		2.6
31	*Cis*-Ocimene				4.0	
32	*trans*-Caryophyllene				1.4	0.7
33	*trans*-Ocimene				0.6	
34	α-Amorphene		8.0			2.0
35	α-Bulnesene					0.2
36	α-Cadinol			0.6	2.1	14.4
37	α-Copaene				0.7	1.3
38	α-Elemol		1.3			0.2
39	α-Gurjunene			0.9		
40	α-Humulene	4.7		2.0	0.6	2.0
41	α-Santalene					2.0
42	α-Ylangene	0.6		0.9		
43	β-Elemene		0.4	3.0	0.7	1.0
44	α-Cadinene	3.6	0.5		0.8	0.4
45	α-Elemene		3.5	1.6		0.5
46	γ-Muurolene				2.6	0.8
47	α-Amosphene			0.8		
48	δ-Cadinene	4.2	0.95	2.6		5.8
49	δ-Cadinol	3.5				1.7
50	τ-Cadinol					8.1
51	9-*epi*-(*E*)-caryophyllene			7.0	1.3	
	*Phenylpropanoids (%)*	0.0	0.0	0.0	61.4	42.3
52	Elemicin				41.7	0.1
53	*E*-asarone				19.7	42.4
54	*Unknown*	0.0	10.0	0.0	0.0	0.0
55	*Unknown*	0.0	0.0	1.2	0.0	0.0
	*Total*	90.1	95.4	90.2	94.4	92.2

**Figure 1.**
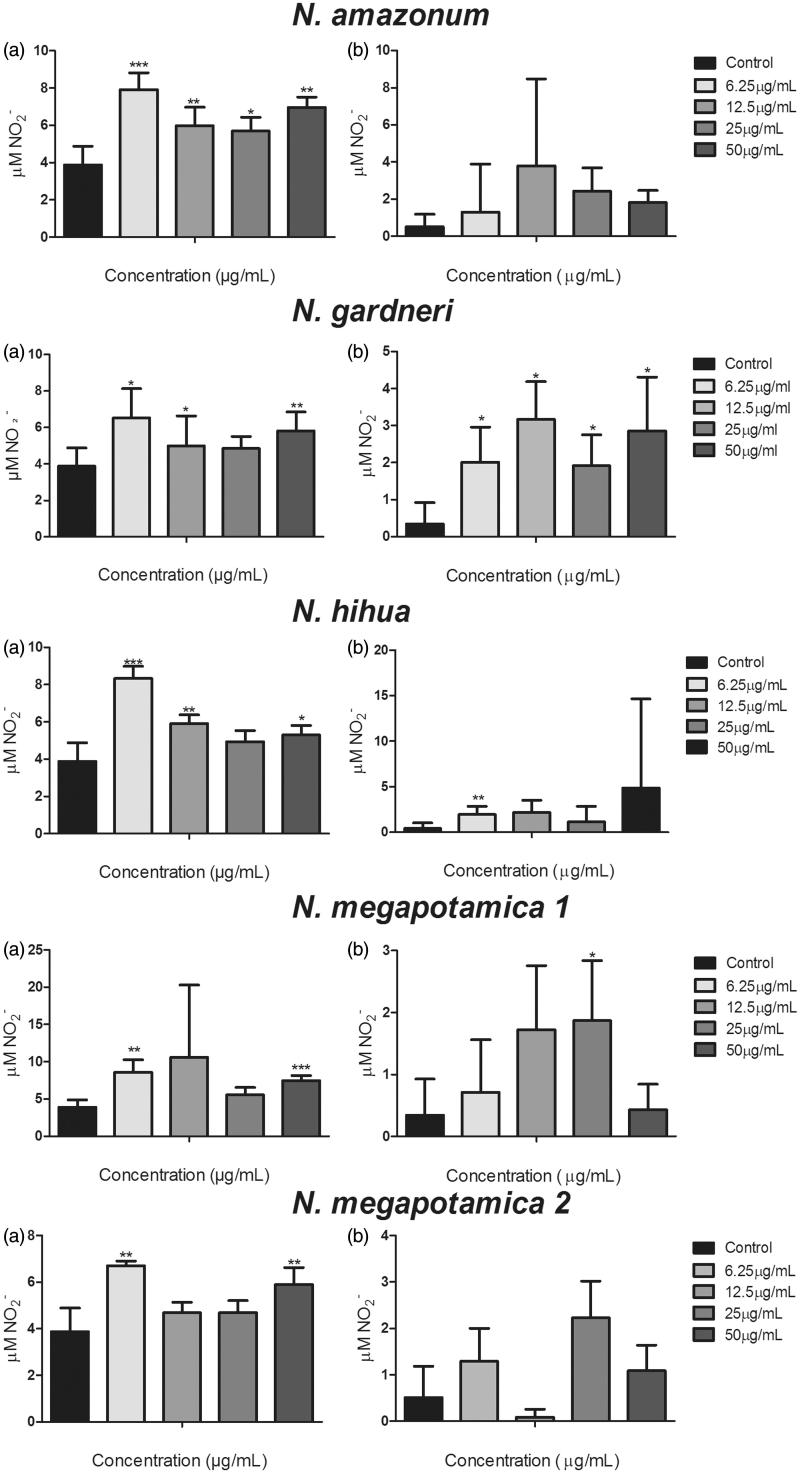
Effect of essential oils from *Nectandra amazonum, N. gardneri*, *N. hihua* and *N. megapotamica* on NO production by peritoneal macrophages infected with *Leishmania amazonensis* (a) and *L. infantum* (b) amastigotes. Bars represent the mean ± SD of six replicates. *p < 0.05, **p < 0.01, ***p < 0.001 for the different concentrations versus untreated cells (control) (Student’s *t* test). The data are representative of three independent experiments.

NO release by infected macrophages was investigated after treatment with essential oils from *Nectandra* spp. (Figure 1). The increase in NO release by *L. infantum*-infected cells after treatment with NHEO did not achieve statistical significance at most of the concentrations tested, showing that this oil probably does not act by this indirect mechanism to control *Leishmania* infection. Instead, this oil might act directly on the parasites. On the other hand, NHEO induced a significant increase in NO production by *L. amazonensis*-infected cells, compared to infected control cells. Interestingly, Siqueira et al. (2011) demonstrated that the bicyclogermacrene-rich oil from *A. coriacea* had a direct action on *L. infantum* and *L. amazonensis* promastigote forms, what could help elucidate its mechanism of action.

The essential oil from *N. gardneri* (NGEO) was active on both *L. infantum* and *L. amazonensis* intracellular amastigotes (IC_50_ = 2.7 ± 1.3 and 2.1 ± 1.06 µg/mL, respectively), with low cytotoxicity (Table 2). This oil was also mainly composed by sesquiterpenes (85.4%), with intermediol as the main component (58.2%) (Table 3). To our knowledge, this is the first report of an essential oil with antileishmania activity with this sesquiterpene as major compound. Unfortunately, another compound of this oil (compound **54**, 10%) could not be identified by the consulted data bank (Table 3).

A significant increase in NO (*p* < 0.05) was found after treatment with all concentrations of NGEO (Figure 1) by the cells infected by both *Leishmania* species, suggesting that the antileishmania activity of NGEO may be associated with this important leishmanicidal mechanism (Olekhnovitch and Bousso 2015).

Essential oils from the two specimens of *N. megapotamica* (NMEO1 and NMEO2) had no cytotoxic effect on the mammalian cells tested (Table 2). The phytochemical profile of NMEO1 and NMEO2 varied considerably (Table 3), reflecting possible differences in the collection sites. Indeed, plants collected in different areas may belong to genotypically diverse populations, with consequent differences in the chemical composition (Garcez et al. 2009), once the biosynthesis of secondary metabolites is highly affected by the environmental conditions (Kutchan 2001). The oil from the first specimen (NMEO1), which was collected in a humid zone near a stream, did not show relevant antileishmania activity on *L. infantum* (> 50.0 ± 1.3 µg/mL), with a moderate activity on *L. amazonensis* (Table 2). The chemical analysis identified two phenylpropanoids (61.4%) as the major compounds, elemicin (41.7%) and *E*-asarone (19.7%). NMEO1 also includes sesquiterpenes (19.1%) and monoterpenes (13.9%) (Table 3). On the other hand, the oil from the second specimen (NMEO2), which was collected in an urbanized dried area, was composed mainly of sesquiterpenes (49.9%), with an absence of monoterpenes (Table 3). The higher concentration of sesquiterpenes may have reflected a water stress condition to which the plant was submitted, and contributed to improving the activity against *L. infantum* intracellular amastigotes (IC_50_ = 12.5 ± 1.4 µg/mL, Table 2). Indeed, Yadav et al. (2014) demonstrated an increase of sesquiterpenes in the essential oil from *Artemisia annua* L. submitted to a prolonged effect of water stress. Interestingly, the phenylpropanoid *E*-asarone was the major compound of NMEO2 (42.4%) and possibly influenced the antileishmania activity. To our knowledge, this is the first report of such an association.

NMEO2, although potentially active on *L. infantum* intracellular amastigotes, did not induce a significant NO release by the host cells (Figure 1). On *L. amazonensis*-infected cells, however, the oils from both *N. megapotamica* specimens induced a significant increase in NO production, which may be mediating the intracellular killing of parasites.

*Nectandra amazonum* essential oil (NAEO) showed moderate activity against *L. infantum* and *L. amazonensis* with a low selectivity index (Table 2), evincing a relevant cytotoxicity. NAEO was composed of 90.1% sesquiterpenes, with a major presence of β-caryophyllene (28.5%). This compound was previously demonstrated to be active against *L. amazonensis* by Carmo et al. (2012) after isolation from *Piper duckei* C.DC. β-Caryophyllene was even more active than the entire essential oil, which contained 27.1% of this compound. Soares et al. (2013) have also demonstrated the anti-*L. amazonensis* activity of *trans*-β-caryophyllene isolated from *Copaifera* spp. commercial oil. On the other hand, Hadri et al. (2010) showed the cytotoxic effect of β-caryophyllene on HCT-116, MCF-7, and murine macrophage RAW264.7 cancer cell lines, thus, likely contributing to the cytotoxic effect found for NAEO in our work. This cytotoxic effect can be reinforced by the fact that NAEO was able to induce a significant increase in NO production by peritoneal cells infected by *L. amazonensis* (Figure 1).

NAEO appears to act independently of NO production by macrophages infected by *L. infantum* (Figure 1). Likewise, Soares et al. (2013) demonstrated that the antileishmania activity of β-caryophyllene (the main compound of NAEO) was not associated with NO production. Carmargos et al. (2014) observed the cytotoxic effect of terpenes (monoterpenes and a sesquiterpene) in fluidity and disruption of the plasma membrane of *L. amazonensis*. Thus, their mechanism of action would be associated with the attack on the plasma membrane of the parasites (restricted to the lipid component, and not to proteins), as already described for terpenes against protozoan, fungal pathogens and tumour cells.

## Conclusions

To our knowledge, this work is the first investigation of the antileishmania activity of the essential oils from *Nectandra* spp. The oils from *N. gardneri* and *N. hihua* showed potential activity and low cytotoxicity on mammalian cells, becoming promising sources of compounds for the development of new antileishmania drugs. Chemical profiles showed a considerable presence of sesquiterpenes, whose isolation is being carried out in order to perform the antileishmania tests. Except for *N. gardneri*, the oils from *Nectandra* seemingly do not stimulate NO production. We propose that future studies should test the direct application of these oils on parasites.

## References

[CIT0001] Alvar J, Vélez ID, Bern C, Herrero M, Desjeux P, Cano J, Jannin J, den Boer M. 2012 Leishmaniasis worldwide and global estimates of its incidence. PLoS One. 7:e35671 22693548 10.1371/journal.pone.0035671 PMC3365071

[CIT0002] Alves FM, Ishii IH. 2007 Lauraceae no município de Corumbá, Mato Grosso do Sul, Brasil. Rodrigésia. 58:179–192.

[CIT0003] Alves FM, Sartori ALB. 2009 *Nectandra* Rol. Ex Rottb. (Lauraceae) no Mato Grosso do Sul, Brasil. Acta Bot Bras. 23:118–129.

[CIT0004] Bohlke M, Guinaudeau H, Angerhofer CK, Wongpanich V, Soerjato DD, Farnsworth NR. 1996 Costaricine, a new antiplasmodial bisbenzylisoquinoline alkaloid from *Nectandra salicifolia* trunk bark. J Nat Prod. 59:576–580. 8786363 10.1021/np960195h

[CIT0005] Bosquiroli LSS, Demarque DP, Rizk YS, Cunha MC, Marques MCS, Matos MFC, Kadri MCT, Carollo CA, Arruda CCP. 2015 *In vitro* anti-*Leishmania infantum* activity of essential oil from *Piper angustifolium*. Rev Bras Farmacogn. 25:124–128.

[CIT0006] Brahmachari G. 2012 Natural products in drug discovery: impacts and opportunities – an assessment In: Brahmachari G, editor. Bioactive natural products: opportunities and challenges in medicinal chemistry. Singapore: World Scientific Publishing Co. Pte. Ltd, p. 1–199.

[CIT0007] Carmargos HS, Moreira RA, Mendanha SA, Fernandes KS, Dorta ML, Alonso A. 2014 Terpenes increase the lipid dynamics in the *Leishmania* plasma membrane at concentrations similar to their IC_50_ values. PLoS One. 9:e104429 25101672 10.1371/journal.pone.0104429 PMC4125203

[CIT0008] Carmo DFM, Amaral ACF, Machado GMC, Leon LL, Silva JRA. 2012 Chemical and biological analyses of the essential oils and main constituents of *Piper* species. Molecules. 17:1819–1829. 22330429 10.3390/molecules17021819 PMC6268953

[CIT0009] Costa EC, Cassamale TB, Carvalho DB, Bosquiroli LSS, Ojeda M, Ximenes TV, Matos MFC, Kadri MCT, Baroni ACM, Arruda CCP. 2016 Antileishmania activity and structure-activity relationship of triazolic compounds derived from the neolignans grandisin, veraguensin, and machilin G. Molecules. 21:802. 10.3390/molecules21060802 PMC6273954 27331807

[CIT0010] Costa EV, Pinheiro MLB, Silva JRA, Maia BHLNS, Duarte MCT, Amaral ACF, Machado GMC, Leon LL. 2009 Antimicrobial and antileishmania activity of essential oil from the leaves of *Annona foetida* (Annonaceae). Quím Nova. 32:78–81.

[CIT0011] Damasceno Júnior GA, Nakajima JN, Rezende UM. 2000 Levantamento florístico das cabeceiras dos rios Negro, Aquidauana, Taquari e Miranda no Pantanal, Mato Grosso do Sul, Brasil In: Willink PW, Chernoff B, Alonso LE, Montambault JR, Lourival R, editors. Uma avaliação biológica dos ecossistemas aquáticos do Pantanal, Mato Grosso do Sul, Brasil. Washington, DC: Conservation International; p. 152–162.

[CIT0012] Ding AH, Nathan CF, Stuer DJ. 1988 Release of reactive nitrogen intermediates and reactive oxygen intermediates from mouse peritoneal macrophages. Comparison of activating cytokines and evidence for independent production. J Immunol. 141:2407–2412. 3139757

[CIT0013] Freitas-Junior LH, Chatelain E, Kim HA, Siqueira-Neto JL. 2012 Visceral leishmaniasis treatment: what do we have, what do we need and how to deliver it? Int J Parasitol: Drugs and Drug Resistance. 2:11–19. 10.1016/j.ijpddr.2012.01.003 PMC3862432 24533267

[CIT0014] Garcez FR, Garcez WS, Hamerski L, Miguita CH. 2009 Fenilpropanoides e outros constituintes bioativos de *Nectandra megapotamica*. Quím Nova. 32:407–411.

[CIT0015] Gottlieb OR. 1972 Review article: chemosystematics of the Lauraceae. Phytochemistiry. 11:1537–1570.

[CIT0016] Hadri A, del Río AG, Sanz J, Coloma AG, Idaomar M, Ozonas BR, González JB, Reus MIS. 2010 Cytotoxic effect of α-humulene and transcaryophyllene from *Salvia officinalis* in animal and human tumor cells. Anal Real Acad Nac Farm. 76:343–356.

[CIT0017] Kutchan TM. 2001 Ecological arsenal and developmental dispatcher. The paradigm of secondary metabolism. Plant Physiol. 125:58–60. 11154296 10.1104/pp.125.1.58 PMC1539325

[CIT0018] Le Quesne PW, Larrahondo JE, Raffaulf RF. 1980 Antitumor plants. X. Constituents of *Nectandra rigida*. J Nat Prod. 43:353–359. 7400821 10.1021/np50009a006

[CIT0019] Machado RRP, Valente Júnior W, Lesche B, Coimbra ES, Souza NB, Abramo C, Soares GLG, Kaplan MAC. 2012 Essential oil from leaves of *Lantana camara*: a potential source of medicine against leishmaniasis. Rev Bras Farmacogn. 22:1011–1017.

[CIT0020] MacRae WD, Towers GHN. 1984 Biological activities of lignans. Phytochemistry. 23:1207–1220.

[CIT0021] Melo JO, Truiti MCT, Muscará MN, Bolonheis SM, Dantas JA, Caparroz-Assef SM, Cuman RKN, Bersani-Amado CA. 2006 Anti-inflammatory activity of crude extract and fractions of *Nectandra falcifolia* leaves. Biol Pharm Bull. 29:2241–2245. 17077521 10.1248/bpb.29.2241

[CIT0022] Monks A, Scudiero D, Skehan P, Shoemaker R, Pau K, Vistica D, Hose C, Langley J, Cronise P, Vaigro-Wolff A, et al 1991 Feasibility of a high-flux anticancer drug screen using a diverse panel of cultured human tumor cell lines. J Natl Cancer Inst. 83:757–766. 2041050 10.1093/jnci/83.11.757

[CIT0023] Monzote L, García M, Montalvo AM, Scull R, Miranda M. 2010 Chemistry, cytotoxicity and antileishmanial activity of the essential oil from *Piper auritum*. Mem Inst Oswaldo Cruz. 105:168–173. 20428676 10.1590/s0074-02762010000200010

[CIT0024] Muñoz V, Sauvain M, Bourdy G, Callapa J, Bergeron S, Roj I, Bravo JA, Balderrama L, Ortiz B, Gimenez A, Deharo E. 2000 A search for natural bioactive compounds in Bolivia through a multidisciplinary approach. Part I. Evaluation of the antimalarial activity of plants used by the Chacobo Indians. J Ethnopharmacol. 69:127–137. 10687869 10.1016/s0378-8741(99)00148-8

[CIT0025] Olekhnovitch R, Bousso P. 2015 Induction, propagation, and activity of host nitric oxide: lessons from *Leishmania* infection. Trends Parasitol. 31:653–664. 26440786 10.1016/j.pt.2015.08.001

[CIT0026] Paladi CS, Pimentel IAS, Katz S, Cunha RLOR, Judice WAS, Caires ACF, Barbieri CL. 2012 *In vitro* and *in vivo* activity of a palladacycle complex on *Leishmania (Leishmania) amazonensis*. PLoS Negl Trop Dis. 6:e1626 22616018 10.1371/journal.pntd.0001626 PMC3352823

[CIT0027] Pott A, Pott VJ, Sciamarelli A, Sartori ALB, Rezende UM, Scremim-Dias E, Jacques EL, Aragaki S, Nakajima JN, Romero R, Cristaldo ACM, Damasceno Júnior GA. 2006 Inventário de Angiospermas do complexo Aporé-Sucuriú In: Pogotto TCS, Souza PR, editors. Biodiversidade do complexo Aporé-Sucuriú: subsídios à conservação e manejo do bioma Cerrado. Campo Grande: Editora UFMS; p. 45–66.

[CIT0028] Ready PD. 2014 Epidemiology of visceral leishmaniasis. Clin Epidemiol. 6:147–154. 24833919 10.2147/CLEP.S44267 PMC4014360

[CIT0029] Rohwer JG. 1993 Lauraceae: *Nectandra*. Flora Neotrop. 60:1–332.

[CIT0030] Rohwer JG, Kubitzki K. 1993 Ecogeographical differentiation in *Nectandra* (Lauraceae), and its historical implications. Bot Acta. 106:88–99.

[CIT0031] Romoff P, Ferreira MJP, Padilla R, Toyama DO, Fávero OA, Lago JHG. 2010 Chemical composition of volatile oils from leaves of *Nectandra megapotamica* Spreng. (Lauraceae). Quím Nova. 33:1119–1121.

[CIT0032] Santos Filho D, Gilbert B. 1975 The alkaloids of *Nectandra megapotamica*. Phytochemistry. 14:821–822.

[CIT0033] Sharifi-Rad J, Sureda A, Tenore GC, Daglia M, Sharifi-Rad M, Valussi M, Tundis R, Sharifi-Rad M, Loizzo MR, Ademiluyi AO, et al 2017 Biological activities of essential oils: from plant chemoecology to traditional healing systems. Molecules. 22:70. 10.3390/molecules22010070 PMC6155610 28045446

[CIT0035] Silva-Filho AA, Albuquerque S, Eberlin MN, Tomazela DM, Bastos JK. 2004 Tetrahydrofuran lignans from *Nectandra megapotamica* with trypanocidal activity. J Nat Prod. 67:42–45. 14738383 10.1021/np0302697

[CIT0034] Silva-Filho AA, Costa ES, Cunha WR, Silva MLA, Dhammika Nanayakkara NP, Bastos JK. 2008 *In vitro* antileishmania and antimalarial activities of tetrahydrofuran lignans isolated from *Nectandra megapotamica*. Phytother Res 22:1307–1310. 18688887 10.1002/ptr.2486

[CIT0036] Siqueira CA, Oliani J, Sartoratto A, Queiroga CL, Moreno PR, Reimão JQ, Tempone AG, Fischer DCH. 2011 Chemical constituents of the volatile oil from leaves of *Annona coriacea* and *in vitro* antiprotozoal activity. Rev Bras Farmacogn. 21:33–40.

[CIT0037] Skehan P, Storeng R, Scudiero D, Monks A, Mcmahon J, Vistica D, Warren JT, Bokesch H, Kenney S, Boyd MR. 1990 New colorimetric cytotoxicity assay for anticancer-drug screening. J Natl Cancer Inst. 82:1107–1112. 2359136 10.1093/jnci/82.13.1107

[CIT0038] Soares DC, Portella NA, Ramos MFS, Siani AC, Saraiva EM. 2013 *trans*-β-Caryophyllene: an effective antileishmania compound found in commercial copaiba oil (*Copaifera* spp.). Evid Based Complement Alternat Med. 2013:761323. 23864897 10.1155/2013/761323 PMC3705974

[CIT0039] Yadav RK, Sangwan RS, Sabir F, Srivastava AK, Sangwan NS. 2014 Effect of prolonged water stress on specialized secondary metabolites, peltate glandular trichomes, and pathway gene expression in *Artemisia annua* L. Plant Physiol Bioch. 74:70–83. 10.1016/j.plaphy.2013.10.023 24269871

